# Inhibition of cancer cell proliferation by 5-fluoro-2'-deoxycytidine, a DNA methylation inhibitor, through activation of DNA damage response pathway

**DOI:** 10.1186/2193-1801-1-65

**Published:** 2012-12-13

**Authors:** Quanyi Zhao, Jiadong Fan, Wei Hong, Lianyun Li, Min Wu

**Affiliations:** Hubei Clinical Centre & Key Laboratory of Intestinal and Colorectal Diseases, and College of Life Sciences, Wuhan University, Wuhan, Hubei, 430072 China

**Keywords:** FCdR, DNA methylation, DNA damage response, Cell cycle, p53, Cancer

## Abstract

Multiple epigenetic changes, including alterations in DNA methylation occur during tumorigenesis. Various inhibitors of DNA methylation have been developed to prevent proliferation of cancer cells. 5-fluoro-2^′^-deoxycytidine (FCdR) is one such DNA methylation inhibitor, which is currently in phase II clinical trial. To investigate the molecular mechanism/s by which FCdR might mediate repression of tumor cell proliferation, we analyzed the toxicity of FCdR in various cell lines established from different sarcomas. We found HCT116, a colon cancer cell line, is much more sensitive to FCdR compared to others. FCdR treatment inhibited HCT116 cells at G2/M check point and up-regulated expression of multiple cancer-related genes, which could be due to its inhibitory activity towards DNA methylation. Furthermore, we found that FCdR activates DNA damage response pathway. Using an inhibitor for ATM and ATR kinases activity, which are required for amplifying the DNA damage repair signal, we show that FCdR induced inhibition of HCT116 cells at G2/M is mediated through activation of DNA damage response pathway.

**Electronic supplementary material:**

The online version of this article (doi:10.1186/2193-1801-1-65) contains supplementary material, which is available to authorized users.

## Background

DNA methylation is a covalent modification of methyl group on the 5C site of cytosine nucleoside (Robertson 2005; Ren et al. 2011) and is dynamically regulated by methylation and demethylation (Carey et al. 2011). High level of DNA methylation on gene promoters is associated with transcriptional repression of genes. During carcinogenesis, global levels of DNA methylation decrease along with progression of cancer. Concomitantly, promoters of tumor suppressors gain DNA methylation, which allow cancer cells to grow unrestrained (Robertson 2005; Esteller 2008; Ellis et al. 2009). These observations have led to the development of small molecule inhibitors capable of inhibiting DNA methylation. These are thought to suppress tumorigenesis by activating the expression of tumor suppressor genes. Some of these DNA methylation inhibitors, such as Vidaza (5-azacytidine, 5-azaC) and Decitabine (5-aza-2^′^-deoxycytidine, 5-azaCdR) have been approved by FDA for treatment of myelodysplatic syndrome (Ellis et al. 2009; Ren et al. 2011). Although many other non-nucleoside DNA methylation inhibitors have been synthesized, their activities in inhibiting DNA methylation and gene activation are relatively weaker and their potential use in clinics still needs to be investigated (Chuang et al. 2005). 5-fluoro-2^′^-deoxycytidine (FCdR, FdCyd) is a well-known DNA methylation inhibitor discovered in early 1990’s and is currently under evaluation in clinical trials of breast cancer and other advanced solid tumors (Gowher and Jeltsch 2004; Ellis et al. 2009; Ren et al. 2011).

Like Vidaza and Decitabine, FCdR is a pyrimidine analogue and can integrate into chromatin, and inhibit DNA methylation (Ellis et al. 2009). Fluorine occupies the 5C site of cytidine, which prevents the modification by methyl group (Ren et al. 2011). Additionally, it was demonstrated that FCdR is capable of binding and trapping DNA methyltransferases, and thus can prevent further DNA methylation (Jones and Taylor 1980; Reither et al. 2003). FCdR was found to be not stable in multiple clinical studies (Beumer et al. 2006), but when combined with other drugs, such as tetrahydrouridine (THU) and dihydro-5-azacytidine (DHAC), FCdR showed increased stability and improved activity (Beumer et al. 2008; Kratzke et al. 2008). However, the molecular mechanism of repression of tumor suppression by FCdR has not been studied in any detail.

Upon treatment with DNA methylation inhibitors, tumor suppressor genes are activated, which then lead to cell cycle arrest or apoptosis. p53 is one of the best characterized tumor suppressor gene, mutated in up to 50% of cancers (Royds and Iacopetta 2006). p53 can be activated by various signals, such as irradiation or chemical induced DNA damage, abnormal oncogene expression, microtubule inhibitors and other stress conditions (Royds and Iacopetta 2006; Kruse and Gu 2009). Upon activation, p53 is phosphorylated and dissociated from MDM2, which results in its stabilization (Wade et al. 2010). Activated p53 transcribes a number of genes to induce cell cycle arrest, apoptosis, and senescence, all of which help in suppressing tumorigenesis (Vousden and Prives 2009; Speidel 2010; Aylon and Oren 2011).

Activation of DNA damage response is one of the most important mechanisms that represses tumorigenesis (Lord and Ashworth 2012). Malignancy of tumor is frequently associated with damage to chromatin, recombination and translocation (Schar 2001). Upon DNA damage, H2AX is phosphorylated by ATM, ATR or DNAPK at the DNA repair sites (Bonner et al. 2008). Phosphorylated H2AX further recruits the above kinases to the damaged foci, which results in amplification of the DNA damage signal (Bonner et al. 2008). ATM and ATR then phosphorylate CHK1, CHK2 and other molecules involved in DNA damage response to arrest cell cycle (Kastan and Lim 2000; Cimprich and Cortez 2008).

In order to investigate the molecular mechanisms of tumor repression by FCdR, we studied its effect on cell fate, gene expression and activation of signaling pathways. We found that FCdR represses proliferation of HCT116 at IC50 between 0.025-0.05 μM. FCdR induced cell cycle arrest at G2/M phase and activated both p53 signaling and DNA damage response pathways. Our results suggest that FCdR induced G2/M arrest and suppression of cancer cell proliferation is mediated through FCdR’s role in activation of DNA repair pathway.

## Results and discussion

### FCdR inhibits proliferation of multiple cancer cell lines

**Figure 1:**
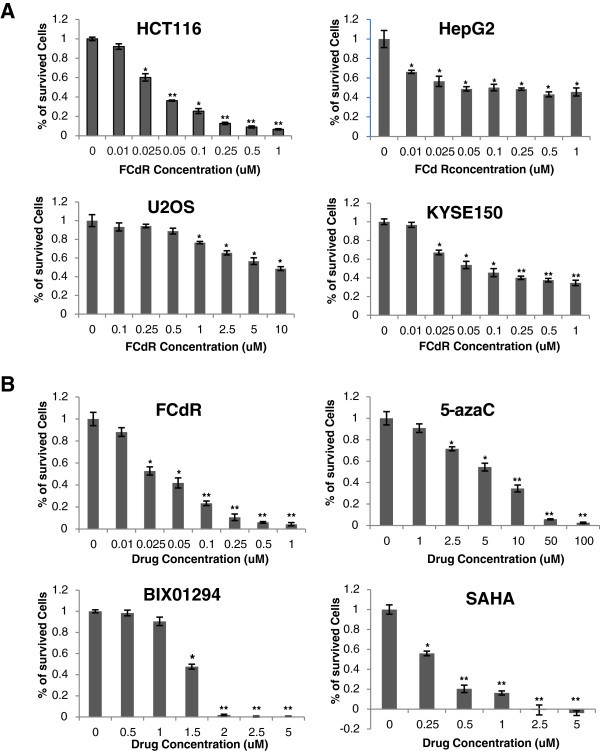
**HCT116 cells are sensitive to FCdR treatment.A**. HCT116, HepG2, U2OS and KYSE150 cell lines were treated with FCdR at indicated concentrations for 72 h and MTT assays performed. HCT116 cells were the most sensitive to FCdR. **B**. Four inhibitors against epigenetic enzymes were used to treat HCT116 cells for 72 h, and MTT assay performed to test their toxicity. HCT116 cells were more sensitive to FCdR and SAHA, compared to 5-azaC and BIX01294. (* p value < 0.05; ** p value < 0.01).

FCdR is in phase II clinical trial for treatment of breast cancer and many solid tumors. In order to test if cancer cells other than breast cancer cells are sensitive to FCdR, we chose HCT116, HEPG2, U2OS and KYSE150 cell lines representing colorectal carcinoma, hepatocellular carcinoma, osteosarcoma and oesophageal squamous cell carcinoma, respectively. We treated these cells with a series of FCdR concentrations. Surviving cells after 72 h treatment were then used to assay by MTT assay. FCdR inhibited the proliferation of all the above cell lines, but to different degrees. HCT116 cells showed less than 10% survival rate with 1 μM FCdR and IC50 was between 0.025-0.05 μM (Figure 1A). At the same 1 μM FCdR concentration, the survival rates of HEPG2, U2OS and KYSE150 cells were about 40%, 80% and 30%, respectively. The observations suggest that colorectal tumors might be more sensitive to FCdR, compared to hepatocellular carcinoma, osteosarcoma and oesophageal squamous cell carcinoma.

### HCT116 cells are more sensitive to FCdR than SAHA and 5-azaC

Several small molecules inhibiting epigenetic processes have been developed with an ability to inhibit cancer cells. SAHA and 5-azaC are two such small molecule inhibitors that have been approved by FDA. We tested and compared the cyto-toxicity of FCdR with SAHA and 5-azaC on HCT116 cells, as well as one novel identified H3K9 methylation inhibitor BIX01294. We found that all the drugs tested repressed the proliferation of HCT116, however, their IC50 differed considerably (Figure 1B). IC50 of FCdR was lowest between 0.025-0.05 μM, whereas for 5-azaC, BIX01294 and SAHA, it was 5 μM, 1.5 μM and 0.25 μM respectively. These findings suggested that HCT116 is much more sensitive to FCdR compared to SAHA and 5-azaC, which may prove to be of value in a clinical study.

### FCdR induces G2/M arrest in HCT116 cell

**Figure 2:**
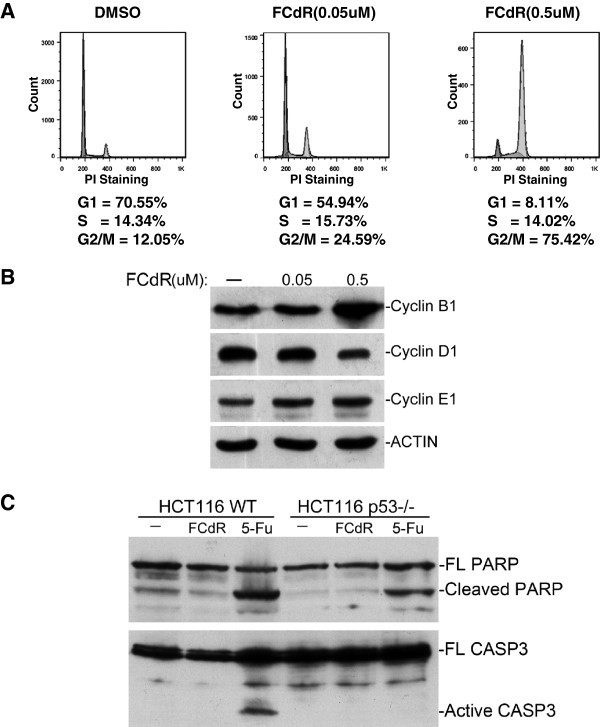
**FCdR arrested HCT116 cells at G2/M phase.A**. HCT116 cells were treated with 0.05 μM or 0.5 μM FCdR for 48 h and cell cycle was analyzed by flow cytometry. Compared with control, percentage of cells at G2/M phase increased significantly after FCdR treatment. **B**. HCT116 cells were treated with indicated amount of FCdR for 48 h and the cyclins checked by western blot. CyclinB1, which accumulates at G2/M phase, significantly increased after FCdR treatment. **C**. HCT116 p53+/+ and p53^−/−^ cells were treated with 0.5 μM FCdR or 375 μM 5-Fu for 48 h and apoptosis assayed by PARP and CASP3 western blot. 5-Fu treatment caused cleavage of PARP and CASP3, but not FCdR.

Next we sought to study the effect of FCdR on cell cycle in HCT116 cells. Since drugs targeting DNA methylation are known to induce cell cycle arrest or apoptosis, we first performed cell cycle analysis by PI (Propidium Iodide) staining and analyzed cells with flow cytometry. Cells treated with 0.05 μM FCdR for 48 h showed upto 24% of cells in G2/M phase (Figure 2A), whereas treatment with 0.5 μM FCdR increased the percentage of cells in the G2M phase to 75% (Figure 2A). These results suggest that FCdR induces G2/M arrest in HCT116. To further substantiate our conclusion, we analysed the expression of cyclins by western blot (Figure 2B). Treatment with 0.5 μM FCdR for 48 h, resulted in significant increase in the total levels of cyclin B1.

Persistent cell cycle arrest leads to induction of apoptosis. However, HCT116 cells treated with FCdR at concentrations of up to 0.5 μM for 48 h, did not show any obvious apoptotic phenotype as observed by light microscopy (data not shown). Flow cytometry analysis of these cells also did not show any obvious sub-G1 peak, which is a characteristic of apoptotic cells (Figure 2A). We further examined the formation of cleaved CASP3 and cleaved PARP, which are hallmarks of apoptosis. We did not detect any cleaved CASP3 or cleaved PARP by western blot whereas 5FU treatment, which induces apoptosis in HCT116 cells, resulted in cleavage of CASP3 and PARP (Figure 2C). These observations suggested that at the given concentration FCdR solely induces G2/M arrest in HCT116 and not apoptosis.

### FCdR alters gene expression pattern by elevating transcription level

**Figure 3:**
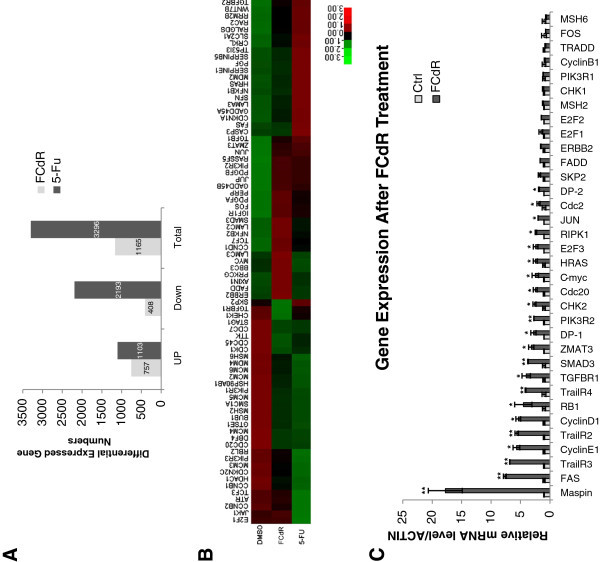
**FCdR induced alteration of global gene expression pattern.** 0.5 μM FCdR or 375 μM 5-Fu were used to treat HCT116 cells for 24 h. Total RNA was used for gene expression pattern analysis by high throughput sequencing. **A**. The number of differentially expressed genes was calculated. FCdR caused changes in expression of 1165 genes, out of which 757 were up-regulated and 408 were down-regulated. 5-Fu caused changes in expression of 3295 genes, out of which 1103 were up-regulated, and 2193 were down-regulated. FCdR-treated cells have a much higher percentage of up-regulated genes. **B**. Differentially expressed genes involved in top 6 pathways (p53 signaling pathway, colorectal cancer, nucleotide excision repair, DNA replication, cell cycle, pathways in cancer) were subjected to cluster analysis. **C**. The change in expression of 45 genes was confirmed by real time PCR. (* p value < 0.05; ** p value < 0.01).

DNA methylation at gene promoters represses transcriptional activation and its inhibitors up regulate expression of genes. To investigate the mechanism/s involved in FCdR-induced G2/M arrest, we performed genome wide RNA sequencing (RNA-seq) of HCT116 cells treated with or without FCdR for 24 h and analyzed the alterations in gene expression. We also performed a similar experiment with 5-Fluorouracil, a widely used chemotherapeutic drug which induces DNA damage and cell cycle arrest, and used the RNA-seq profile for comparison with FCdR dataset. To reduce background signals we only considered genes, expressions of which were changed by at least two fold. We found that FCdR treatment lead to alteration in expression of a total of 1165 genes, out of which 757 were up regulated and 408 were down regulated (Figure 3A and Additional file 1: Table S1). A higher number of up-regulated genes in FCdR treated cells is expected as FCdR is known to inhibit DNA methylation. In comparison, 5-Fu treatment resulted in change in expression of 3296 genes out of which, 2/3 were down regulated (Figure 3A and Additional file 2: Table S2).

Next we looked at alterations of signaling pathways, and found many of them to be altered in cells treated with FCdR (Table 1). The pathways, which were significantly altered were also related with cancer, including p53 signaling, DNA repair, DNA replication, cell cycle (Table 1). We validated the altered expression of 45 genes involved in these pathways by reverse transcription followed by quantitative PCR (Figure 3C). We found that more than 90% of these genes were similarly altered as in our high throughput sequencing dataset.

**Table 1:** **Top 20 pathways altered in FCdR treated cells**

#	Pathway	DEGs with pathway annotation (594)	All genes with pathway annotation (9952)	Pvalue	Qvalue	Pathway ID
1	p53 signaling pathway	16 (2.69%)	67 (0.67%)	1.38E-06	1.56E-04	ko04115
2	Colorectal cancer	14 (2.36%)	62 (0.62%)	1.26E-05	9.54E-04	ko05210
3	Nucleotide excision repair	12 (2.02%)	48 (0.48%)	1.75E-05	9.94E-04	ko03420
4	DNA replication	10 (1.68%)	36 (0.36%)	3.28E-05	1.49E-03	ko03030
5	Cell cycle	20 (3.37%)	128 (1.29%)	6.57E-05	2.49E-03	ko04110
6	Pathways in cancer	36 (6.06%)	323 (3.25%)	2.06E-04	6.00E-03	ko05200
7	Limonene and pinene degradation	3 (0.51%)	3 (0.03%)	2.12E-04	6.00E-03	ko00903
8	Biosynthesis of secondary metabolites	26 (4.38%)	225 (2.26%)	8.97E-04	2.26E-02	ko01110
9	Metabolic pathways	92 (15.49%)	1132 (11.37%)	1.07E-03	2.43E-02	ko01100
10	Benzoate degradation	4 (0.67%)	9 (0.09%)	1.24E-03	2.57E-02	ko00362
11	Mismatch repair	6 (1.01%)	23 (0.23%)	1.86E-03	3.26E-02	ko03430
12	Prostate cancer	13 (2.19%)	88 (0.88%)	2.05E-03	3.26E-02	ko05215
13	Cell cycle - yeast	11 (1.85%)	68 (0.68%)	2.13E-03	3.26E-02	ko04111
14	Bladder cancer	8 (1.35%)	40 (0.4%)	2.16E-03	3.26E-02	ko05219
15	Meiosis - yeast	10 (1.68%)	59 (0.59%)	2.35E-03	3.33E-02	ko04113
16	Ribosome	13 (2.19%)	90 (0.9%)	2.52E-03	3.36E-02	ko03010
17	Adherens junction	11 (1.85%)	70 (0.7%)	2.70E-03	3.41E-02	ko04520
18	Base excision repair	7 (1.18%)	34 (0.34%)	3.41E-03	4.04E-02	ko03410
19	Naphthalene degradation	2 (0.34%)	2 (0.02%)	3.56E-03	4.04E-02	ko00626
20	Focal adhesion	22 (3.7%)	199 (2%)	3.76E-03	4.06E-02	ko04510

**Figure 4:**
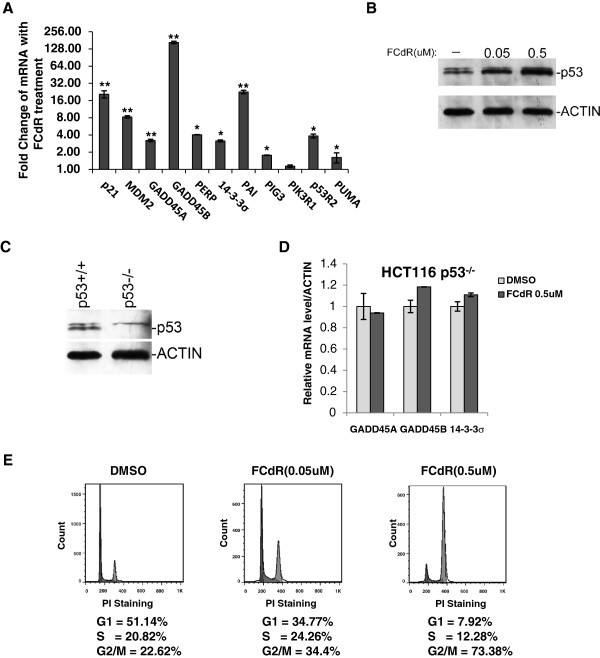
**FCdR treatment activates p53 pathway.A**. Differentially expressed genes of p53 signaling pathway found in high throughput RNA sequencing were confirmed by real time PCR. **B**. HCT116 wild type cells were treated with indicated amounts of FCdR for 24 h and p53 was analyzed by western blot. **C**. HCT116 p53^+/+^ and p53^−/−^ cells were verified by western blot. **D**. p53^−/−^ cells were treated with 0.5 μM FCdR for 24 h and expression of indicated p53 downstream genes assayed by real time PCR. These genes were not activated with p53 deficiency. **E**. p53^−/−^ cells were treated with FCdR for 48 h and cell cycle was analyzed. FCdR was still able to induce G2/M arrest without p53. (* p value < 0.05; ** p value < 0.01).

We performed cluster analysis of differentially expressed genes involved in pathways, which were altered the most, including- p53 signaling pathway, colorectal cancer, nucleotide excision repair, DNA replication, cell cycle, pathways in cancer. We observed that both FCdR and 5-Fu treatment lead to similar changes in genes involved in DNA replication, DNA damage repair and p53 pathway (Figure 3B). Expression of a number of genes involved in DNA replication and repair were reduced in cells with both drugs. p53 target genes such as MDM2, CDKN1A/p21, SFN/14-3-3σ, and SERPINE1/PAI were also found to be activated in both samples, though in comparison to FCdR, 5-Fu treatment resulted in stronger up-regulation of these p53 targets (Figures 3B and 4A). Among the genes up-regulated by FCdR, we also found several well-known proto-oncogenes, such as HRAS, CMYC and ERBB2 (Figure 3B and C). Increased expression of these genes might have implications in cancer treatment. Interestingly, we also observed that the receptor of TRAIL, TRAILR2, and the two decoy receptors, TRAILR3 and TRAILR4, were overexpressed (Figure 3C). TRAIL is a potential drugable protein which is known to induce apoptosis in many cancer cell lines but not in normal cells. It will be interesting to look at the effect of cancer treatment combining FCdR with TRAIL.

### FCdR treatment activated p53 signaling pathway in HCT116

Our gene expression analysis of FCdR treated HCT116 cells suggest that FCdR activates p53 signaling pathway, which is the most important pathway inhibiting tumorigenesis. We further tested and confirmed the activation of p53 pathway by RTPCR analysis of mRNA levels of p53 target genes. We tested 11 p53 downstream genes and found that all were significantly elevated in expression (Figure 4A). As the activation of p53 involves stabilization of p53 protein, we analysed and found that the amount of p53 protein significantly increased after FCdR treatment, combined with the discovery that multiple p53 target genes increased their expression, suggesting that FCdR probably activates p53 pathway (Figure 4B).

**Figure 5:**
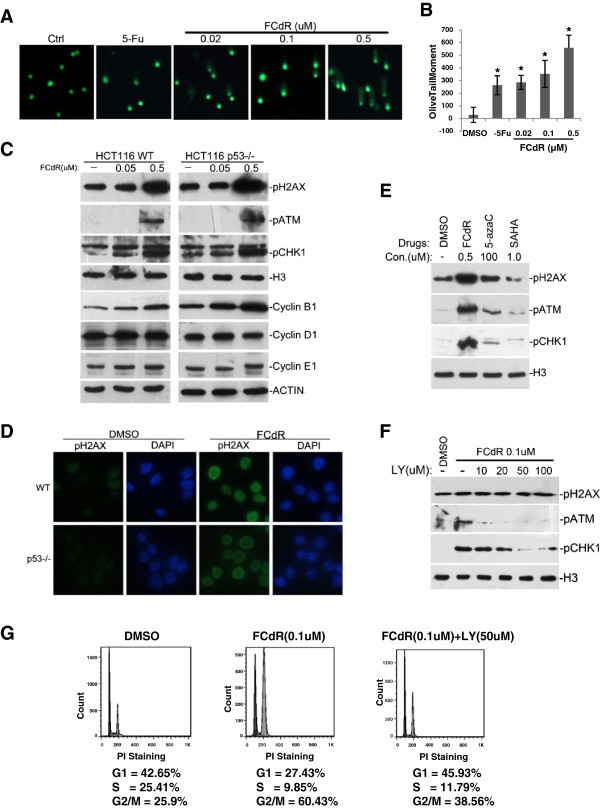
**DNA damage response is responsible for FCdR-induced cell cycle arrest.A**. Cells were treated with indicated amount of chemicals for 12 h and damaged DNA was detected by alkaline comet assay. **B**. Olive tail moment in the previous assay was calculated according to manufacturer’s method and the statistic results were shown. **C**. HCT116 p53^+/+^ and p53^−/−^ cells were treated with FCdR. Markers for DNA damage response (pH2AX, pATM and pCHK1) and cell cycle (Cyclin B1, Cyclin D1 and Cyclin E1) were analyzed by western blot. Histone H3 and β-ACTIN were used as loading controls. **D**. HCT116 p53^+/+^ and p53^−/−^ cells were treated with FCdR for 8 h and immunofluorescence staining was performed to show FCdR induced H2AX phosphorylation in both cell lines. **E**. Three drugs were used to treat HCT116 for 8 h and DNA damage responses were investigated by western blotting. FCdR and 5-azaC were able to induce phosphorylation of H2AX, ATM and CHK1, but not SAHA. H3 was used as control. **F**. The inhibitory effect of LY294002 to FCdR induced DNA damage response was assayed. **G**. Addition of 50 μM LY294002 restored the G2/M arrest induced by 0.1 μM FCdR. (* p value < 0.05).

In order to investigate if p53 signaling pathway is responsible for cell cycle arrest caused by FCdR treatment, we performed FCdR treatment in a p53 kncokout HCT116 cell line. We first verified the absence of p53 protein in these cells by western blot (Figure 4C). These cells, when treated with FCdR at a concentration of 0.5 μM, did not activate p53 target genes, including GADD45A, GADD45B and 14-3-3σ (Figure 4D). To our surprise, FCdR was still able to induce G2/M arrest in these cells in the absence of p53 (Figure 4E). Compared with parental HCT116 cells (Figure 2A), these cells showed G2/M arrest and similar distribution profile of other phases of cell cycle Also, cyclin B1 accumulation was comparable to parental cells (Figure 5A). Taken together, above observations suggest that the G2M arrest observed in FCdR treated cells is not a consequence of activation of the p53 pathway.

### FCdR induces DNA damage response in HCT116

Since FCdR is capable of integrating into chromatin, and our FCdR treatment of HCT116 cells activated transcription of genes involved in DNA repair (Table 1), we investigated if FCdR has a role in DNA damage response pathway. To assess it, we first performed alkaline comet assay, and found that HCT116 cells treated with a low concentration of 0.02 μM FCdR for 12 h exhibited DNA damage similar with 100 μM 5-Fu, and the extent of DNA breaks increases at increasing doses of FCdR (Figure 5A and B). We then tested for phosphorylation of H2AX, ATM and CHK1, which are hallmarks of activated DNA repair pathway, and occur early during the DNA repair response. Western blot results showed a dramatic increase in levels of phosphorylated H2AX, ATM and CHK1 in HCT116 cells treated with 0.5 μM FCdR (Figure 5C). Immunofluorescent staining also showed accumulation of phosphorylated H2AX in the nuclei of FCdR treated HCT116 cells (Figure 5D). Since it is well known that activation of DNA damage response causes cell cycle arrest, it is highly likely that activation of DNA repair pathway is the primary reason of FCdR-induced cell cycle arrest.

To test if the induction of DNA damage response is a common feature for DNA methylation inhibitors, we treated HCT116 cells with various drugs, including two inhibitors of DNA methylation, FCdR and 5-azaC, and a histone deacetylase inhibitor SAHA. We observed that FCdR and 5-azaC treatment increased levels of phosphorylated H2AX, ATM and CHK1, whereas SAHA treatment did not show a significant increase (Figure 5E). This indicated that at least two DNA methylation inhibitors, FCdR and 50azaC, are able to activate DNA damage pathway at the indicated concentration.

To confirm if DNA damage response is the primary reason for FCdR induced cell cycle arrest, we investigated if addition of a small molecule LY294002, an inhibitor of DNA damage response can suppress the activation of FCdR mediated DNA damage response pathway. LY294002 inhibits the activity of multiple PI3K kinases, including ATM and ATR, the two key kinases involved in DNA damage response. Various combinations of different concentrations of FCdR and LY294002 were tested.We found that at concentrations higher than 50 μM, LY294002 inhibits phosphorylation of ATM and CHK1 induced by 0.1 μM FCdR (Figure 5F). We performed cell cycle analysis on cells treated with both FCdR and LY294002, and compared with cells treated only with FCdR. We found that G2/M arrest observed in cells treated with 0.1 μM FCdR was completely abolished in cells treated additionally with DNA damage response inhibitor LY294002 (Figure 5G). This observation suggests that FCdR induced G2/M arrest is mediated through activation of DNA damage response pathway.

## Conclusions

The inhibitors of DNA methylation and histone deacetylation have shown similar curative effects and reduced toxicity, compared to traditional chemotherapy drugs in treatment of cancers. To speed up their use in cancer treatment, it is critical to elucidate the cellular response and molecular mechanisms of these drugs. FCdR is a promising drug in clinical trial. However, we know little about the kinds of tumors which are sensitive to FCdR and the molecular mechanisms of FCdR mediated suppression of tumorigenesis. We found that HCT116, a colon cancer cell line, was extremely sensitive to FCdR, which suggested that FCdR might be effective in treatment of certain types of colon cancer. FCdR inhibits HCT116 proliferation by arresting cell cycle at G2/M phase, without activating the apoptotic pathway. By global gene expression profiling we found that p53 signaling is activated upon FCdR treatment. Interestingly, FCdR induced cell cycle arrest was not dependent on the activation of p53 pathway. Many chemotherapy drugs induce death in cancer cells through p53 activation; however, since p53 is mutated in more than 50% cancers, the curative effects of chemotherapy drugs vary among patients. FCdR might be useful in treating tumors with mutation in p53 gene.

Our results show that FCdR treatment causes global changes in gene expression in HCT116 cells, which may help us better understand the molecular mechanisms of FCdR-induced cellular responses. Not only had we observed up regulation of tumor suppressor genes, such as p21 and PUMA, we also observed increase of HRAS and CMYC, two well known oncogene. It will be important to evaluate their roles in FCdR-induced response. Compared with 5-Fu, FCdR caused less genes to express differentially but a higher percentage of upregulated genes. The ability of FCdR to inhibit DNA methylation may explain the observation that most altered genes were upregulated in FCdR treated cells.

FCdR also activated DNA damage response pathway, possibly due to its ability to incorporate into chromatin. Since, an inhibitor of ATM/ATR kinases, LY294002, can rescue the cell cycle arrest induced by FCdR, it suggested the activation of ATM/ATR pathways is responsible for the observed cell cycle arrest. It is likely that FCdR inhibits cell growth primarily by activating the DNA damage response pathway. The activation of p53 is an important consequence of DNA damage response. FCdR induced cell cycle arrest is not dependent on p53 activation, which suggests other molecules downstream of DNA damage pathway might be responsible. Another inhibitor of DNA methylation, 5-azaC also induced DNA damage response, but not SAHA, an inhibitor of histone deacetylation. It will be interesting to investigate if DNA damage response is a common mechanism involved in growth inhibition caused by DNA methylation inhibitors.

## Materials and methods

### Cell lines, antibodies and reagents

FCdR, 5-azaC, 5-azaCdR and BIX01294 were purchased from Sigma. SAHA (Vorinostat) was purchased from Cayman. HCT116 and U2OS cells were purchased from ATCC. KYSE150 was purchased from Cell Bank of Chinese Academy of Medical Science. HepG2 was a gift from Dr. Jianguo Wu (Wuhan University). HCT116 p53^−/−^ cell was a gift from Dr. Pengfei Wang of Stowers Institute for Medical Research. The antibodies against Cyclin B1 (Epitomics), Cyclin D1 (Epitomics), Cyclin E1 (Epitomics), p-H2AX (Epitomics), p-ATM (Epitomics), p-CHK1 (Epitomics), β-ACTIN (CWBIO), CASP (Cell Signaling) and H3 (Abcam), were purchased from indicated companies. Rabbit anti-PARP was a gift from Dr. Xiaodong Zhang (Wuhan University). Rabbit anti-p53 was raised in our lab against purified full length protein. The PCR primers are given in Additional file 3: Table S3.

### MTT assay

Cells were split at 1×10^3^ cells per well in 96-well plate. After 24 h cells were treated with drugs and cultured for 72 h. 25 μg MTT was then added to each well and cells incubated for 4 h at 37°C. The medium with the formazan sediment was dissolved in 50% DMF and 30% SDS (pH4.7). The absorption was read at 570nM. P value was calculated by *t* test.

### Cell cycle assay

Cells were split at 2-3×10^5^cells per well in 6-well plates. After 12-14 h cells were treated with drugs and cultured for 48 h. Cells were harvested by 0.05% Trypsin-EDTA digestion and centrifuged after PBS wash. Cells were fixed overnight with 70% ethanol. Flow cytometry analysis was performed after PI staining (50ug/mL) and RNase digestion (100ug/mL) at 37°C for 30 min.

### Western blot

Approximately 2 × 10^6^ Cells were lyzed in 200ul 1×SDS loading buffer () and boiled at 95°C for 10 min. 5-10 μl sample was loaded to SDS PAGE gel for each lane and the separated proteins were transferred to nitrocellular membrane. The membrane was blocked in 5% milk and hybridized with indicated first antibody over night and second antibody for 1 h before developing.

### Immuno-fluorescence staining

Cells were cultured on cover slips, washed twice with PBS and then fixed with chilled methanol. Cells were then washed three times with PBS and blocked in PBS with 1% BSA for 10 min. Cells were incubated with primary and secondary antibodies for one hour, respectively. Samples were mounted with prolong anti-fade kit (Invitrogen) and observed on a fluorescent microscope.

### Reverse transcription and quantitative PCR

Cells were scraped and collected by centrifugation. Total RNA was extracted with RNA extraction kit (Yuanpinghao) according to manufacturer’s protocol. Approximately 1ug of total RNA was used for reverse transcription with a first strand cDNA synthesis kit (Toyobo). The amount of mRNA was assayed by quantitative PCR. β-actin was used to normalize the amount of each sample. Assays were repeated at least three times. Data shown were average values ± SD of one representative experiment. P value was calculated by *t* test.

### Alkaline comet assay

OxiSelect Comet assay kit was purchased from Cell Biolabs and comet assay was performed according to the manufacturer’s protocol. Briefly, cells were split at 2-3×10^5^ cells per well in 6-well plate and cultured for 12 h. Drugs were added to the medium and cells were treated for indicated time. Individual cells are mixed with molten agarose and then treated with lysis buffer and alkaline solution. Following electrophoresis, the samples were dried and stained with a DNA dye, then observed with fluorescent microscope. The tail length of each cell was measured manually and the tail DNA percentage was quantified by using Quantity One software (Bio-rad). Then the Olive tail moment was calculated according to the following formula: Tail DNA% X Tail moment length, as suggested by provided manual. Data shown were average values ± SD. P value was calculated by *t* test.

### Next generation sequencing and data analysis

The cells were treated with desired drugs for 24 h before collection. Total RNA was extracted and reverse transcribed. Then the cDNA were analyzed by BGI. To study the relationship of the differential expressed genes, the values of selected genes were submitted for cluster analysis by using Cluster3.0 and the heatmap was presented by Java Treeview.

## Electronic supplementary material

Additional file 1: **Table S1.** Differential Expressed Genes between FCdR-treated and Control Cells. (XLS 512 KB)

Additional file 2: **Table S2.** Differential Expressed Genes between 5-Fu-treated and control cells. (XLS 56 KB)

Additional file 3: **Table S3.** Information of primers used in the study. (XLS 56 KB)
